# Interventional Approach to Left Main Coronary Artery Dissection

**DOI:** 10.7759/cureus.3410

**Published:** 2018-10-04

**Authors:** Rabih Tabet, Nikhil Nalluri, Farshid Daneshvar, James Malpeso

**Affiliations:** 1 Internal Medicine, Staten Island University Hospital / Northwell Health, Staten Island, USA; 2 Cardiology, Staten Island University Hospital / Northwell Health, Staten Island, USA

**Keywords:** left main coronary artery dissection, angioplasty complication, interventional cardiology

## Abstract

A left main coronary artery (LMCA) iatrogenic dissection is a rare but potentially life-threatening complication of coronary angioplasty. It can range from a simple tear in the artery wall to a severe dissection, causing complete blood flow obstruction. We report the case of a 63-year-old male patient who was presented to our catheterization laboratory following a positive stress test. An angiogram showed a proximal left anterior descending (LAD) artery tight lesion. Balloon inflation was complicated by an ostial LAD dissection that rapidly extended into the left main and the left circumflex arteries treated with angioplasty and stenting. Cardiac catheterization four days later showed a residual LMCA intimal flap that remained asymptomatic and stable. This is an interesting case of a stable LMCA dissection with the intimal flap intermittently obstructing the ostium of the left anterior descending artery. In addition, we will discuss the factors that increase the risk of coronary dissection and focus on methods to help prevent the occurrence of such complications.

## Introduction

Coronary artery dissection is one of the most feared complications that can occur during a coronary intervention. Some dissections can be simple and do not mandate any intervention since they heal on their own (Types A & B in the National Heart, Lung, and Blood Institute [NHLBI] classification system for coronary artery dissection) [1]. Other dissections can be more severe, causing complete blood flow obstruction and leading to drastic outcomes unless they were rapidly and optimally managed (Types C through F) [1]. The incidence of iatrogenic left main coronary artery (LMCA) dissection is generally low (approximately 0.07%), but it mandates immediate intervention due to the unpredictable course of the dissection flap that could lead to the deterioration of the antegrade blood flow and hemodynamic instability [1-2]. Here, we present a rare case of an iatrogenic retrograde LMCA dissection that remained stable for four days without causing symptoms or complications.

## Case presentation

**Figure 1:**
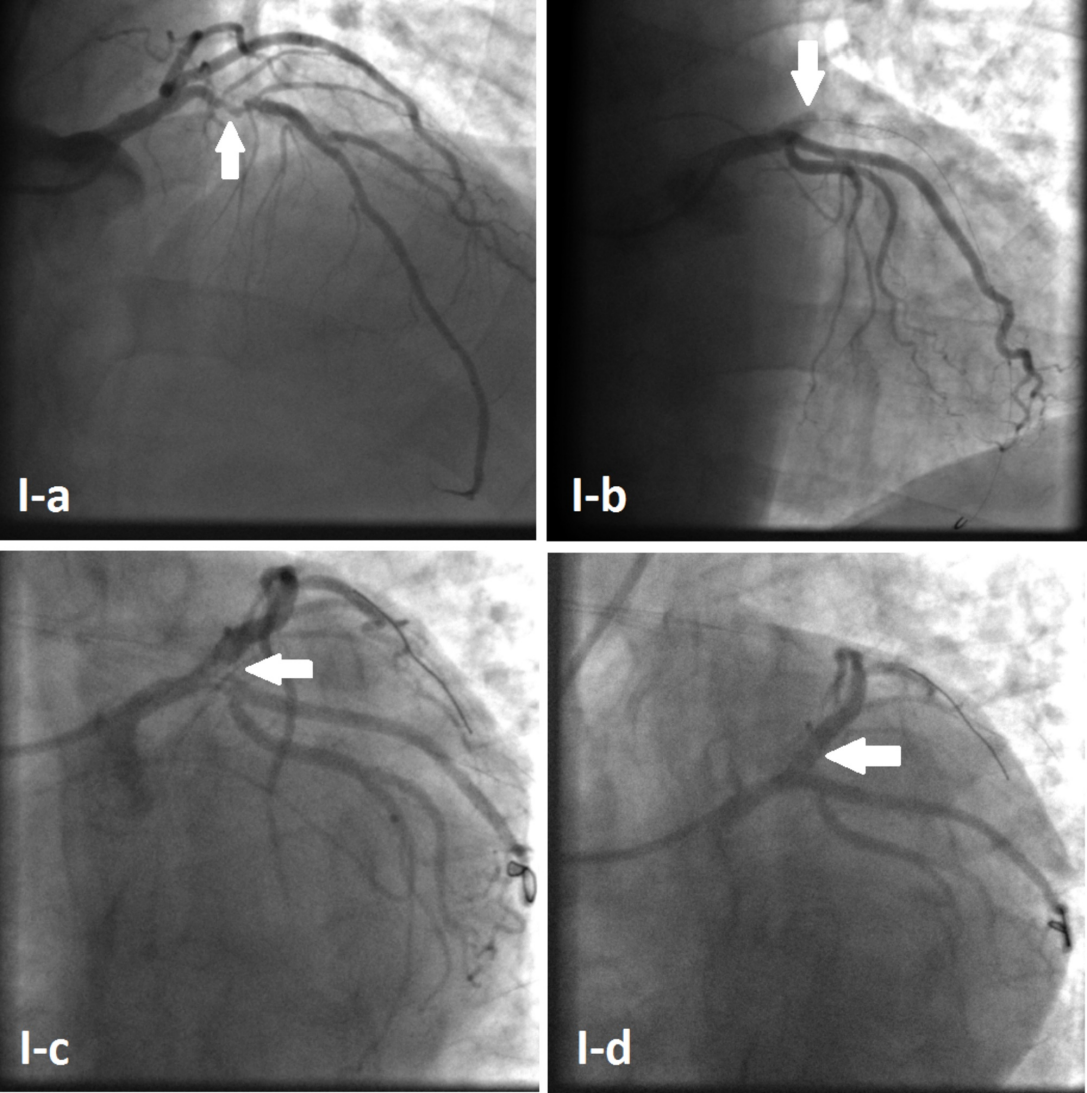
Initial percutaneous coronary intervention Coronary angiogram showing proximal LAD tight lesion (Arrow I-a). Complete obstruction of blood flow in the LAD artery (Arrow I-b). Retrograde dissection extending to the LMCA and the LCx (Arrow I-c). Haziness at the proximal LAD giving the impression of blood clots, with preserved distal flow (Arrow I-d). LAD:  left anterior descending  artery; LMCA:  left main coronary artery; LCx: left circumflex artery.

A 63-year-old man presented to our institution in order to undergo elective cardiac catheterization following a positive stress test that was done because of recurrent episodes of atypical chest pain. The patient is previously known to have hypercholesterolemia only. He used to take no medications. He never smoked cigarettes or used illicit drugs, but admits moderate alcohol consumption. Baseline electrocardiogram (ECG) and two-dimensional (2D) echocardiography were within the normal range. Coronary angiography was then performed via the right radial artery using a 5F Judkins left 3.5 diagnostic catheter, which showed a tight lesion at the proximal left anterior descending (LAD) coronary artery (Figure 1a) that was pre-dilated with a 3.0x12 Mav2 RX balloon with a maximum inflation pressure of 12 atm. The following angiogram showed a proximal LAD type F dissection with complete blood flow obstruction (Figure 1b), with an extension of the flap to the LMCA and the left circumflex (LCx) artery (Figure 1c). Immediate angioplasty with stenting was performed successfully at the bifurcation of the distal LMCA with the LAD and the LCx (Figure 1d) using the V technique. A 3.5x15 Onyx drug-eluting stent (Medtronic, Minneapolis, US) inflated to 12 atm was used for the LMCA-LAD lesion, and a 3.0x12 Onyx drug-eluting stent (Medtronic, Minneapolis, US), again inflated to 12 atm, was used for the ostial LCx lesion.

**Figure 2:**
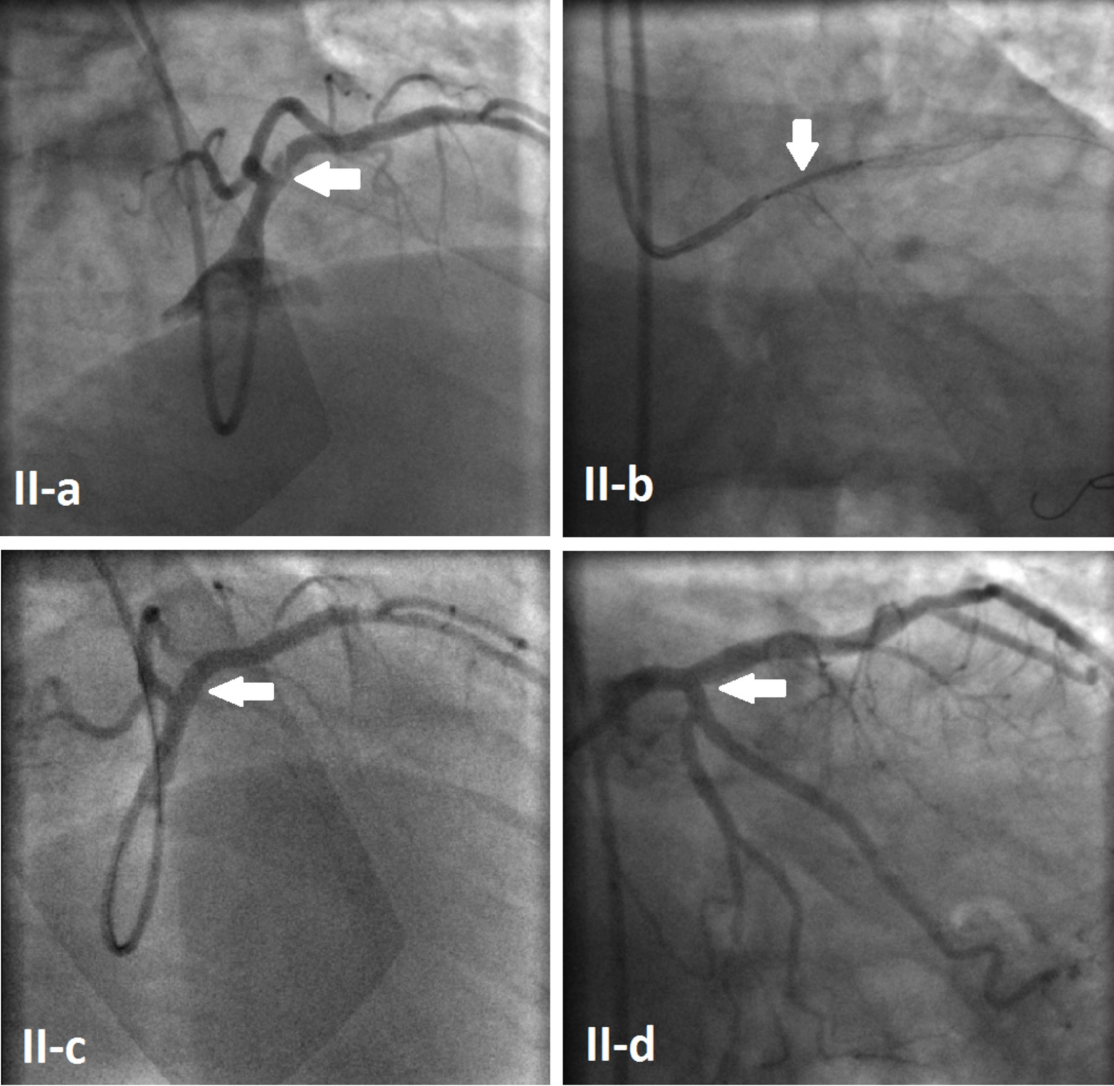
Follow-up coronary angiogram Floating intimal flap extending from the distal LMCA to the proximal LAD (Arrow II-a). Angioplasty with stent placement from the distal LMCA to the LAD (Arrow II-b). Final angiogram showing complete treatment of the dissection (Arrows II-c & II-d). LAD:  left anterior descending  artery; LMCA:  left main coronary artery; LCx: left circumflex artery.

The final angiogram showed a thrombolysis in the myocardial infarction (TIMI) III flow to both the LAD and LCx, but minimal haziness was noted around the ostium of the LAD, giving the impression of some blood clots in the area. The decision was taken to admit the patient to the coronary care unit (CCU) and treat him with intravenous eptifibatide to dissolve the LAD clot. The patient remained asymptomatic in the CCU without any chest pain, rising troponins, or changes in surface ECG. Four days later, the patient was taken back to the cardiac catheterization laboratory for a second look. An angiography revealed a distal LMCA intimal flap, extending into the proximal LAD (Figure 2a). Although it was a type-B dissection, the intimal flap was floating at the ostium of the LAD, causing intermittent obstruction of the blood flow, so an angioplasty with stenting was performed from the distal LMCA to the proximal LAD (Figure 2b) using a 3.5x15 Onyx drug-eluting stent inflated to 12 atm. This led to the restoration of normal blood flow (Figures 2c-2d). The patient was discharged home two days later without any complication.

## Discussion

Complications following a coronary angioplasty are quite common and can range from minor incidents that are self-limited, to more severe and lethal complications. Coronary artery dissection, intramural hematoma, perforation, distal embolization, side branch occlusion, failure of stent deployment, acute kidney injury, stroke, and access site bleeding, among many others, constitute the various complications that can occur during a coronary angioplasty procedure [3]. Iatrogenic LMCA dissections are rare during percutaneous coronary interventions but may be catastrophic if not immediately recognized and managed. Various factors can increase the risk of developing an LMCA dissection such as significant atherosclerosis, inappropriate catheter selection, deep intubation, forceful manipulation of the guide catheter, forceful injection of the contrast medium, the use of stiff coronary guidewires, and an inexperienced operator, among others [4]. To the best of our knowledge, this is the first case of a distal LMCA dissection with the intimal flap intermittently covering the ostium of the LAD and remaining asymptomatic and stable for more than four days. Such dissections are usually managed depending on four criteria: i) presence of clinical symptoms, ii) hemodynamic status, iii) ECG changes, and iv) coronary flow distal to the affected segment [2]. Although our patient did not have any of these four criteria, we decided to proceed with the angioplasty and stenting of the dissected segment for multiple reasons. The patient’s condition may not have deteriorated because he was receiving intravenous eptifibatide which prevented thrombus formation and extension at the site of the dissection. The patient did not have anginal symptoms or ECG changes because the intimal flap was only intermittently obstructing the blood flow through the LAD and did not cause constant obstruction. Finally, we had a high degree of suspicion that this dissection may worsen once the eptifibatide is stopped leading to rapid thrombus formation and complete obstruction of the proximal LAD causing sudden hemodynamic compromise and possible death.

Current data does not offer a clear consensus or guidelines for the treatment of LMCA dissections. But since such dissections have an unpredictable course and can have fatal outcomes, we keep the final decision of treating with prompt revascularization or conservatively to the operator at the time of the event. In the end, the prevention of complications is preferable to treating them. Some of the risk factors for iatrogenic LMCA dissection are non-modifiable such as the extent of the left main atherosclerosis and the variant anatomy of the coronary ostia [4]. But other factors are modifiable and should be addressed, such as the presence of an experienced operator, the choice of guide catheter and handling techniques (limiting catheter manipulation and avoiding deep LMCA intubation), avoiding the use of stiff or hydrophilic guidewires, use of low-pressure balloon predilatation, avoiding oversized balloons or stents, and finally, avoiding forceful contrast injection especially if the pressure is damped [4-5].

## Conclusions

In conclusion, iatrogenic LMCA dissection constitutes a dynamic condition that requires prompt recognition and management. Knowledge of the predisposing factors, familiarity with the angiographic appearance, and skilled operators are required for avoiding or treating this potentially catastrophic complication should it arise. Finally, a decision for immediate revascularization versus watchful waiting should be tailored on a case-by-case manner.
